# Accelerated dimensionality reduction of single-cell RNA sequencing data with fastglmpca

**DOI:** 10.1093/bioinformatics/btae494

**Published:** 2024-08-07

**Authors:** Eric Weine, Peter Carbonetto, Matthew Stephens

**Affiliations:** Laboratory for Information and Decision Systems, Massachusetts Institute of Technology, Cambridge, MA 02139, United States; Department of Data Science, Dana Farber Cancer Institute, Boston, MA 02215, United States; Department of Human Genetics, University of Chicago, Chicago, IL 60637, United States; Department of Human Genetics, University of Chicago, Chicago, IL 60637, United States; Department of Statistics, University of Chicago, Chicago, IL 60637, United States

## Abstract

**Summary:**

Motivated by theoretical and practical issues that arise when applying Principal component analysis (PCA) to count data, Townes *et al.* introduced “Poisson GLM-PCA”, a variation of PCA adapted to count data, as a tool for dimensionality reduction of single-cell RNA sequencing (scRNA-seq) data. However, fitting GLM-PCA is computationally challenging. Here we study this problem, and show that a simple algorithm, which we call “Alternating Poisson Regression” (APR), produces better quality fits, and in less time, than existing algorithms. APR is also memory-efficient and lends itself to parallel implementation on multi-core processors, both of which are helpful for handling large scRNA-seq datasets. We illustrate the benefits of this approach in three publicly available scRNA-seq datasets. The new algorithms are implemented in an R package, fastglmpca.

**Availability and implementation:**

The fastglmpca R package is released on CRAN for Windows, macOS and Linux, and the source code is available at github.com/stephenslab/fastglmpca under the open source GPL-3 license. Scripts to reproduce the results in this paper are also available in the GitHub repository and on Zenodo.

Almost every analysis of single-cell RNA sequencing (scRNA-seq) data involves some kind of dimensionality reduction to help summarize and denoise the data (Stuart *et al.* 2019, Sun *et al.* 2019, Amezquita *et al.* 2020, Tsuyuzaki *et al.* 2020, Linderman 2021). Principal component analysis (PCA) is a widely used dimensionality reduction technique, but it has been criticized as being poorly suited to the sparse count nature of scRNA-seq data. Motivated by this, Townes *et al.* (2019) suggested instead using a version of PCA, called “GLM-PCA,” that is specifically tailored to count data. However, GLM-PCA is computationally challenging to fit. In this paper we provide faster algorithms, implemented in the software fastglmpca, to fit this model.

The GLM-PCA model combines PCA with ideas from generalized linear models (GLMs) (McCullagh 1989) and dates back at least to Choulakian (1996); see also Collins *et al.* (2001) and Chen *et al.* (2013). Here we consider the Poisson version of this model, which was the primary focus in Townes *et al.* (2019). The Poisson variant models the *n *×* m* data matrix **Y** as

(1) $$\begin{array}{c} y_{ij}∼\text{Pois}(\lambda_{ij})\\ \;\text{log}\;\lambda_{ij}=h_{ij}\\ H=UV^{T}, \end{array}$$

where Pois(λ) denotes the Poisson distribution with mean *λ*; *y_ij_* and *h_ij_* denote entries of the matrices **Y** and **H**, respectively; $U\in R^{n\times K}$ and $V\in R^{m\times K}$ are the matrices of unknowns to be estimated from the data; and *K *>* *0 is an integer specifying the dimension of the reduced representation, typically a number much smaller than *n* or *m*. In this form, the model is symmetric in the rows and columns of **Y**, but by convention we assume that the rows are genes and the columns are cells (e.g. Stuart *et al.* 2019, Townes *et al.* 2019, Nicol and Miller 2023). See the Supplementary Text for elaborations of this model with options to specify row and column covariates.

In Equation (1), we do not impose the typical PCA constraints enforcing orthogonality on **U** and **V** because such constraints can easily be applied after fitting; i.e. once **U, V** have been estimated, a “PCA-like” decomposition for **H** can be obtained from a singular value decomposition of the estimated $UV^{T}$. See the Supplementary Text for details.

Whereas standard PCA involves straightforward application of a (truncated) SVD algorithm, fitting the GLM-PCA model is much less straightforward; computing a maximum-likelihood estimate (MLE) of **U, V** in Equation (1) is a high-dimensional, nonconvex optimization problem. The glmpca R package (Townes *et al.* 2019) uses stochastic gradients, progressively improving the parameter estimates in the direction of a noisy estimate of the gradient computed with a random subset (a “mini-batch”) of the cells. [The R package NewWave fits a related model via stochastic gradients; see (Agostinis *et al.* 2022).] Since the performance of the stochastic gradients method can depend strongly on the choice of learning rate, glmpca used the adaptive AvaGrad method (Savarese *et al.* 2021). However, even with AvaGrad, the method can be very sensitive to the choice of learning rate, and may be unstable if the learning rate is too large. [glmpca implements other approaches, but we and Nicol and Miller (2023) have found that the AvaGrad approach generally performed best.]

The scGBM R package (Nicol and Miller 2023) takes a different approach, iteratively solving an approximation to the log-likelihood that has the form of a more tractable “weighted SVD” problem. This approach, called IRSVD (“iteratively reweighted SVD”), can be very memory-intensive—e.g. it involves forming a matrix of the same size as **Y** that is not sparse—which limits its application to larger scRNA-seq datasets (see also Lee and Han 2022 for a related discussion of these issues).

Algorithm 1 Sketch of Alternating Poisson Regression for GLM-PCA. Row *i* and column *j* of **Y** are denoted, respectively, by $y_{:,i}$ and $y_{:,j}$.

**Require:** Count data $Y\in R_{+}^{n\times m}$, initial estimates $U\in R^{n\times K},\;V\in R^{m\times K}$, and a function Pois_GLM_MLE (X,y) that returns the MLE of β in the Poisson GLM (see Supplementary Text).

1:  **while** not converged **do**

2:   **for**i=1,…,n**do** ▹ These can be performed in parallel.

3:     $u_{i}\leftarrow \;\mathrm{Pois}\_\_\mathrm{GLM}\_\_\mathrm{MLE}(V,y_{i,:}^{T})$

4:    Store $u_{i}$ in the *i*th row of **U**.

5:   **end for**

6:   **for**j=1,…,m**do** ▹ These can be performed in parallel.

7:     $v_{j}\leftarrow \;\mathrm{Pois}\_\_\mathrm{GLM}\_\_\mathrm{MLE}(U,y_{:,j})$

8:     Store $v_{j}$ in the *j*th row of **V**.

9:   **end for**

10:  **end while**

11:  **return U, V**

Here we describe another approach to fitting GLM-PCA models that is based on a simple observation: when **V** is fixed, computing the MLE for **U** reduces to the much simpler and well-studied problem of independently fitting *n* GLMs with a Poisson error distribution and log-link function (McCullagh 1989). Similarly, when **U** is fixed, computing the MLE of **V** reduces to independently fitting *m* Poisson GLMs. This suggests a block-coordinate optimization approach (Wright 2015) that alternates between optimizing **U** with fixed **V**, and optimizing **V** with fixed **U** (Algorithm 1). This approach is analogous to the “alternating least squares” algorithm for truncated SVD (Hastie *et al.* 2015), and a similar alternating approach has proven very effective for nonnegative matrix factorization. We call our approach “Alternating Poisson Regression” (APR) to draw attention to its two key aspects: (i) the alternating optimization of **U** and **V**, and (ii) the reduction to simpler Poisson GLM optimization problems. We have implemented the APR algorithm in the R package fastglmpca.

The APR approach has several benefits. First, it has strong convergence guarantees; the block-coordinatewise updates monotonically improve the log-likelihood, and under mild conditions converge to a (local) maximum of the likelihood (Wright 2015). In addition, by splitting the large optimization problem into smaller pieces (the Poisson GLMs), the computations are memory-efficient and are trivially parallelized to take advantage of multi-core processors.

Since APR reduces to the problem of fitting many (much smaller) Poisson GLMs, the speed of the APR algorithm depends critically on how efficiently one can fit the individual Poisson GLMs. The “classic” algorithm for GLMs is iteratively reweighted least squares (IRLS) (McCullagh 1989). However, the complexity of IRLS grows very quickly with *K*—we would prefer an approach that is also fast for large *K*. Therefore, we instead take a cyclic coordinate descent (CCD) approach to fitting each Poisson GLM, which involves very simple (and therefore very fast) 1-d Newton updates of the GLM parameters. Although the convergence behavior of CCD is theoretically much worse than IRLS, in practice CCD typically converges very quickly, especially when we orthogonalize **U** and **V** at each iteration. These and other implementation details are described in the Supplementary Text.

To illustrate the benefits of the APR approach for GLM-PCA, we analyzed three scRNA-seq datasets: 7193 cells from the tracheal epithelium in wild-type mice (Montoro *et al.* 2018); and two peripheral blood mononuclear cell (PBMC) datasets with 68 579 cells (“68k PBMC”) and 94 655 cells (“95k PBMC”) (Zheng *et al.* 2017). We compared APR, implemented in the R package fastglmpca, and two existing software implementations (also in R): the IRSVD algorithm implemented in scGBM (Miller and Carter 2010, Nicol and Miller 2023) and the AvaGrad algorithm implemented in glmpca (Townes *et al.* 2019, Savarese *et al.* 2021).

Since all the methods optimize the same objective function (the log-likelihood), we used this objective function to compare the quality of the fits. The quality of the fit and the running time depends very strongly on the criterion used to stop the model fitting, and since this criterion is somewhat arbitrary, we performed these comparisons by visualizing the evolution of the log-likelihood over time. The results for different settings of *K*, ranging from 2 to 25, are summarized in Supplementary Figs S1 and S2, and for *K *=* *10 in Fig. 1A. (Full details of these comparisons are given in the Supplementary Text.) The typical result was that while all the algorithms continued to (slowly) improve the fits even after running for many hours, the APR algorithm improved the fit at a much greater rate than the other approaches. (In two exceptions to this, AvaGrad seemed to have settled into better local solutions than APR; Supplementary Fig. S1.) Examining the log-likelihood for each cell reveals that fastglmpca consistently improved the fit across almost all cells, rather than just a small subset of cells (Supplementary Figs S3 and S4). When multi-processor computing resources were available, APR leveraged these resources to dramatically speed up model fitting. For example, to achieve the same log-likelihood as running AvaGrad for 10 h, the parallel APR updates running on a 28-core processor needed to run only 10 min on the 68k PBMC data and only 1 min on the epithelial airway dataset (Fig. 1A).

**Figure 1.**
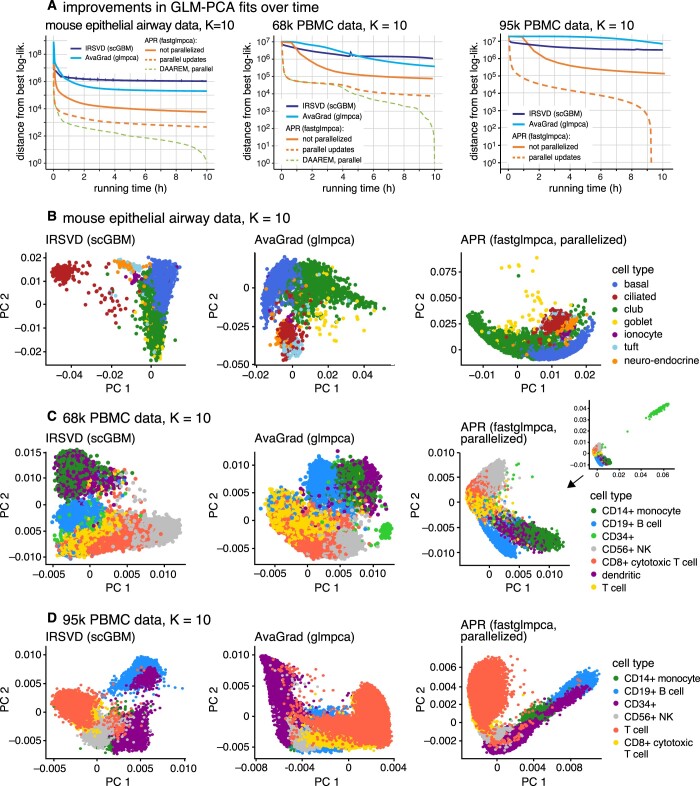
Comparison of GLM-PCA algorithms on three scRNA-seq datasets: mouse epithelial airway data (7193 cells), 68k PBMC data (68 579 cells), and 95k PBMC data (94 655 cells). (A) Improvement in *K *=* *10 GLM-PCA fits over time. Log-likelihoods are shown relative to the best log-likelihood recovered among methods compared. The *Y* axis has a log scale, and log-likelihood differences <1 are shown as 1. (B–D) The first two rows of **V** after fitting the model for about 10 h. The cell types in B and C are estimates from scRNA-seq data (Zheng *et al.* 2017, Montoro *et al.* 2018); the cell types in D are the sorted cell populations (Zheng *et al.* 2017)

To assess whether the different log-likelihoods achieved by different methods corresponded to qualitatively different representations of the data, we examined the estimated latent factors (the “PCs”) returned by each method. We found that the different methods sometimes generated quite different representations; consider, e.g. the results in Fig. 1B–D (see also Supplementary Figs S5–S7). Subjectively, the APR estimates seemed to produce more striking visual representations of the key cell population substructures, but since there was no ground truth we could not say definitively whether one representation was better than the other. The 95k PBMC dataset however did contain an independent ground truth of sorts since it was made up of sorted cell populations (Fig. 1D; Supplementary Text). Therefore, we performed a downstream clustering analysis on each estimate of **V**, and then, following Sun *et al.* (2019), used two measures—the normalized mutual information (NMI) and the adjusted Rand index (ARI)—to assess how well the clusters recovered the sorted cell types (Supplementary Fig. S8). The parallelized APR updates very quickly produced clusters that closely recovered the sorted cell populations, whereas AvaGrad took much longer to obtain clusters of similar accuracy. IRSVD also produced reasonably good clusters quickly, but then seemed to get “stuck”, and had trouble making further improvements.

All algorithms have a computational expense that grows at most linearly in *n*, *m*, and *K* (Supplementary Fig. S9). However, fastglmpca can cope with much larger scRNA-seq datasets because of the care taken to avoid computations that “fill in” the sparse data matrix **Y**. In summary, the key differentiating factors are: (i) the speed at which fastglmpca finds good GLM-PCA fits, particularly when the updates can be run in parallel; (ii) numerical computations that greatly reduce memory usage when the data matrix **Y** is large and sparse. We also observed, anecdotally, the potential to additionally accelerate the model fitting using DAAREM (Henderson and Varadhan 2019) (Fig. 1A).

The Poisson GLM-PCA model (1) can be seen as combining a Poisson measurement model with a low-rank (log) expression model (in the terminology of Sarkar and Stephens 2021). There is good theoretical and empirical support for the Poisson measurement model, but the expression model would likely be improved by allowing for deviations from an exact low-rank structure (Sarkar and Stephens 2021). This idea can motivate alternative models, such as the negative binomial variation of GLM-PCA that is implemented in glmpca (see also the NewWave package; Agostinis *et al.* 2022.) Future work could consider extending the algorithms introduced here to the negative binomial case.

In summary, we contribute a new R package, fastglmpca, which implements fast algorithms for dimensionality reduction of count data based on the Poisson GLM-PCA model. The package is available on CRAN for all major computing platforms. It features a well-documented, user-friendly interface that aligns closely with glmpca and scGBM, including a vignette illustrating its application to scRNA-seq data. The interface splits the GLM-PCA analysis into two phases: an initialization phase, where modeling choices are made, including the rank, *K*, and row- and column-covariates (function “init_glmpca_pois”); and a model fitting phase, where the optimization may be monitored and fine-tuned (function “fit_glmpca_pois”). The core model fitting routines were implemented efficiently in C++ using the Armadillo linear algebra library and Intel Threading Building Blocks (TBB).

## Supplementary Material

btae494_Supplementary_Data

## Data Availability

All data used in this article are publicly available. Please see section 3 of the Supplementary data for complete information regarding data access and pre-processing.
